# Analysis of Patients with *Helicobacter pylori* Infection and the Subsequent Risk of Developing Osteoporosis after Eradication Therapy: A Nationwide Population-Based Cohort Study

**DOI:** 10.1371/journal.pone.0162645

**Published:** 2016-09-14

**Authors:** Hong-Mo Shih, Tai-Yi Hsu, Chih-Yu Chen, Cheng-Li Lin, Chia-Hung Kao, Chao-Hsien Chen, Tse-Yen Yang, Wei-Kung Chen

**Affiliations:** 1 Department of Emergency Medicine, China Medical University Hospital, Taichung, Taiwan; 2 School of Medicine, College of Medicine, China Medical University, Taichung, Taiwan; 3 Management Office for Health Data, China Medical University Hospital, Taichung, Taiwan; 4 Graduate Institute of Clinical Medical Science and School of Medicine, College of Medicine, China Medical University, Taichung, Taiwan; 5 Department of Nuclear Medicine and PET Center, China Medical University Hospital, Taichung, Taiwan; 6 Department of Bioinformatics and Medical Engineering, Asia University, Taichung, Taiwan; 7 Department of Medical Laboratory Science and Biotechnology, China Medical University, Taichung, Taiwan; 8 Molecular and Genomic Epidemiology Center, China Medical University Hospital, China Medical University, Taichung, Taiwan; University of Delhi—South Campus, INDIA

## Abstract

**Purpose:**

Previous studies have reported conflicting results on the association between *Helicobacter pylori* infection and osteoporosis. A few studies have discussed the influence of *H*. *pylori* eradication therapy on bone mineral density.

**Methods:**

We assessed the prevalence of osteoporosis among the *H*. *pylori*-infected population in Taiwan and the influence of early and late *H*. *pylori* eradication therapy on bone mineral density.

**Results:**

Using data from Taiwan's National Health Insurance Research Database, we identified 5,447 patients who received *H*. *pylori* eradication therapy from 2000 to 2010 and 21,788 controls, frequency-matched according to age, sex, and year of receiving *H*. *pylori* eradication therapy. Those who received *H*. *pylori* eradication therapy were divided into two groups based on the time interval between the diagnosis of a peptic ulcer and commencement of eradication therapy. The risk of developing osteoporosis was higher in the early *H*. *pylori* treatment cohort (hazard ratio [HR] = 1.52, 95% confidence interval [CI] = 1.23–1.89) and late *H*. *pylori* treatment cohort (HR = 1.69, 95% CI = 1.39–2.05), compared with the risk in the control cohort. When followed for less than 5 years, both the early and late cohorts had a higher risk of developing osteoporosis (HR = 1.69, 95% CI = 1.32–2.16 and HR = 1.72, 95% CI = 1.38–2.14). However, when the follow-up period was over 5 years, only the late eradication group exhibited a higher incidence of osteoporosis (HR = 1.62, 95% CI = 1.06–2.47).

**Conclusion:**

The development of osteoporosis is complex and multi-factorial. Via this population-based cohort study and adjustment of possible confounding variables, we found *H*. *pylori* infection may be associated with an increased risk of developing osteoporosis in Taiwan. Early eradication could reduce the influence of *H*. *pylori* infection on osteoporosis when the follow-up period is greater than 5 years. Further prospective studies are necessary to discover the connection of *H*. *pylori* and osteoporosis.

## Introduction

Osteoporosis is a common, but difficult to detect, disease among elderly people and is characterized by decreased bone mineral density. The World Health Organization claimed that osteoporotic fractures account for 2.8 million disability-adjusted life years annually in Americans and Europeans. Previous studies have reported age, sex, cigarette smoking, low body mass index, steroid use, and chronic alcohol consumption [1–4] as risk factors for osteoporosis.

Gastrointestinal diseases are also believed to contribute to the development of osteoporosis [5]. Both Tovey et al. and Bisballe et al. observed decreased bone mineral density in post-gastrectomy patients [6, 7]. Previous studies have also described an association between celiac disease and osteoporosis [8]. Inflammatory bowel disease was also suspected of increasing the risk of osteoporosis [9]. Recently, peptic ulcers and atrophic gastritis have been suspected of being risk factors for osteoporosis [10, 11].

Research suggests *Helicobacter pylori* infection is associated with several gastrointestinal diseases, such as chronic gastritis, peptic ulcers, gastric cancer, and mucosa-associated lymphoid tissue lymphoma [12–16]. Recent reports have described the investigation into the extra-gastric effects of *H*. *pylori*, including coronary artery disease, chronic kidney disease, dementia, and anemia [17–19].

Whether *H*. *pylori* infection is a risk factor for osteoporosis remains controversial [20–23]. However, only a few studies have discussed the influence of *H*. *pylori* eradication therapy on bone mineral density. Since *H*. *pylori* infection is the most common chronic bacterial infection of the human upper gastrointestinal tract [24, 25], people who received *H*. *pylori* eradication therapy may have different durations of chronic *H*. *pylori* infection. We suspected that early *H*. *pylori* eradication therapy could minimize the influence of chronic *H*. *pylori* infection on bone mineral density. We conducted a population-based retrospective cohort study by using records from the Taiwan National Health Insurance Research Database (NHIRD) to investigate the incidence of osteoporosis among people who received *H*. *pylori* eradication therapy and the influence of early and late *H*. *pylori* eradication therapy on bone.

## Materials and Methods

### Data Source

A retrospective cohort study was assembled using data from the Longitudinal Health Insurance Database 2000 (LHID2000) provided by Taiwan’s National Health Research Institute (NHRI), which includes information on outpatient, ambulatory, and hospital inpatient care as well as dental services. The National Health Insurance (NHI) program was launched on March 1, 1995 and covered almost 99% of the 23 million residents of Taiwan [26]. The details of the program and the LHID2000 have been adequately described previously [27, 28]. The diagnostic codes in the current study were based on the International Classification of Diseases, Ninth Revision, Clinical Modification (ICD-9-CM). The study was approved by the Institutional Review Board (IRB) of the China Medical University and Hospital (CMUH104-REC2-115).

### Sampled Participants

We recruited patients who were aged 20 years or above who had been diagnosed with peptic ulcer disease (ICD-9-CM codes 531, 532, and 533) and subsequently received *H*. *pylori* eradication therapy. According to the insurance benefits of the NHI system, *H*. *pylori*-related treatments are confirmed with gastroscopy and biopsy. *H*. *pylori* eradication treatment with triple or quadruple therapy was defined as involving multiple differential medicinal treatments: a proton pump inhibitor or H2 receptor blocker, clarithromycin or metronidazole, and amoxicillin or tetracycline, with or without bismuth (details of all eligible *H*. *pylori* eradication regimens have been reported previously) [29]. These drug combinations were prescribed in the same order, and the duration of therapy was between 7 and 14 days. Patients who received *H*. *pylori* eradication therapy within 1 year of diagnosis of peptic ulcer disease were included in the early eradication cohort. Patients who received *H*. *pylori* eradication therapy after 1 year of being initially diagnosed were included in the late eradication cohort. The index date for patients was set as the date that they first received *H*. *pylori* eradication therapy. The exclusion criteria were missing data regarding date of birth, sex, or history of osteoporosis (ICD-9-CM codes 733.0–733.1) before the index date. Controls were randomly selected from the pool of participants without peptic ulcer disease who did not receive *H*. *pylori* eradication therapy. Four controls were frequency-matched to each *H*. *pylori* eradication case according to age (every 5 y span), sex, and year of receiving *H*. *pylori* eradication therapy.

### Outcome and Variables of Interest

The general diagnostic process of the NHI program was based on physical examination and quantitative ultrasound. Diagnosis in partial patients was confirmed using dual energy x-ray absorptiometry following the guidance issued by the Health Promotion Administration, Ministry of Health and Welfare in Taiwan on osteoporosis diagnosis and treatment. All patients were followed until a diagnosis of osteoporosis was made or they were censored for loss to follow-up, withdrawal from the NHI program, or December 31, 2011, whichever occurred first. Coronary artery disease (CAD) (ICD-9-CM codes 410–414), alcohol-related illness (ICD-9-CM codes 291, 303, 305, 571.0, 571.1, 571.2, 571.3, 790.3, A215, and V11.3), stroke (ICD-9-CM codes 430–438), chronic obstructive pulmonary disease (COPD) (ICD-9-CM codes 491, 492, 496), asthma (ICD-9-CM code 493), rheumatoid arthritis (ICD-9-CM code 714), and steroid use were considered as covariates.

### Statistical Analysis

The chi-square test and Student’s *t*-test were used to determine differences in categorical and continuous variables between *H*. *pylori* eradication (including early and late *H*. *pylori* eradication therapy) and control cohorts. The cumulative incidence of osteoporosis among the three cohorts was plotted using the Kaplan–Meier method, and the difference was tested using a log-rank test. The incidence density rate of osteoporosis was calculated for each instance of early *H*. *pylori* eradication therapy, late *H*. *pylori* eradication therapy, and for the control cohort. The incidence rate ratios of the *H*. *pylori* eradication cohorts to that of the control cohort and the 95% confidence interval (CI) were estimated using a Poisson regression model. Multivariable Cox proportional hazard regression analysis was performed to estimate the relative hazard ratios (HRs) and 95% CIs of osteoporosis development for the *H*. *Pylori* eradication cohorts adjusted for age, sex, and comorbidities of CAD, alcohol-related illness, stroke, COPD, asthma, rheumatoid arthritis, and steroid use. All data analyses were performed using the SAS statistical package (Version 9.4 for Windows; SAS institute, Inc., Cary, NC, USA). A 2-tailed *P*<0.05 indicated statistical significance.

## Results

In this study, 5,447 patients who received *H*. *pylori* eradication therapy—comprising 2,721 who received early *H*. *pylori* eradication therapy and 2,726 who received late *H*. *pylori* eradication therapy—and 21,788 controls were investigated (Table 1). Among the three cohorts, most patients were male (62.6% in the early *H*. *pylori* eradication cohort, 59.2% in the late *H*. *pylori* eradication cohort, and 60.9% in the control cohort, separately). The mean age was 51.1±14.3 years in the *H*. *pylori* eradication cohorts and 50.6±14.7 in the control cohort. Both the early and late *H*. *pylori* eradication cohorts exhibited a higher prevalence of CAD, alcohol-related illness, COPD, asthma, rheumatoid arthritis, and steroid use compared with the control cohort (*P*<0.05). The mean follow-up periods were 5.91±3.10 years in the early *H*. *pylori* eradication cohort, 5.07±2.77 years in the late *H*. *pylori* eradication cohort, and 5.52±2.96 years in the control cohort. After 12 years of follow-up, the cumulative incidence of osteoporosis was higher in the early and late *H*. *pylori* eradication cohorts than in the control cohort (*P*<0.001 and *P*<0.001, respectively).

**Table 1:** Comparison of demographics and comorbidity between gastric disease with H pylori eradication and controls.

	Control (N = 21788)		Early HP Eradication (N = 2721)		Late HP Eradication (N = 2726)		Total (N = 5447)		
	n	%	n	%	n	%	n	%	p-value
**Age, year**									0.99
≤49	10560	48.5	1518	55.8	1122	41.2	260	48.5	
50–65	7228	33.2	834	30.7	973	35.7	1807	33.2	
≥65	4000	18.4	369	13.6	631	23.2	1000	18.4	
Mean (SD) ^#^	50.6	14.7	48.5	13.9	53.6	14.3	51.1	14.3	0.03
**Sex**									0.99
Female	8528	39.1	1019	37.5	1113	40.8	2132	39.1	
Male	13260	60.9	1702	62.6	1613	59.2	3315	60.9	
**Comorbidity**									
CAD	2038	9.35	286	10.5	647	23.7	933	17.1	<0.001
Alcohol-related illness	673	3.09	132	4.85	216	7.92	348	6.39	<0.001
Stroke	621	2.85	64	2.35	116	4.26	180	3.30	0.08
COPD	1449	6.65	200	7.35	483	17.7	683	12.5	<0.001
Asthma	957	4.39	127	4.67	288	10.6	415	7.62	<0.001
Rheumatoid arthritis	26	0.12	6	0.22	7	0.26	13	0.24	0.04
**Medication**									
Steroid used	820	3.76	117	4.30	269	9.87	386	7.09	<0.001

Chi-square test compared to total gallstone

^#^Two sample t-test.

The incidence density rates were 6.09, 9.84, and 4.69 per 1000 person-years in the early *H*. *pylori* eradication cohort, late *H*. *pylori* eradication cohort, and control cohort, respectively (Table 2).

**Table 2:** Hazard ratios of Osteoporosis between gastric disease with late HP Eradication and control subjects as well as gastric disease with early HP Eradication and control subjects stratified by demographics and comorbidity.

	Control (N = 21788)		Early HP Eradication (N = 2721)		IRR *	Adjusted HR^†^	Late HP Eradication (N = 2726)		IRR*	Adjusted HR^†^
	Case	Rate^#^	Case	Rate^#^	(95% CI)	(95% CI)	Case	Rate^#^	(95% CI)	(95% CI)
**All**	564	4.69	98	6.09	1.30 (1.16, 1.45)***	1.52 (1.23, 1.89)***	136	9.84	2.10 (1.91, 2.31)***	1.69 (1.39, 2.05)***
**Gender**										
Female	369	7.89	60	10.0	1.27 (1.07, 1.50)**	1.43 (1.09, 1.88)**	79	14.5	1.83 (1.57, 2.13)***	1.63 (1.27, 2.10)***
Men	195	2.65	38	3.77	1.42 (1.23, 1.64)***	1.76 (1.24, 2.49)**	57	6.82	2.57 (2.28, 2.90)***	1.76 (1.29, 2.40)***
**Age**										
≤49	82	1.31	20	2.08	1.59 (1.36, 1.86)***	1.62 (0.99, 2.65)	18	2.91	2.23 (1.89, 2.62)***	1.80 (1.06, 3.06)*
≥50	482	8.38	78	12.1	1.44 (1.24, 1.67)***	1.41 (1.11, 1.79)**	118	15.4	1.84 (1.63, 2.09)***	1.54 (1.25, 1.90)***
**Comorbidity**^**‡**^										
No	366	3.68	59	4.67	1.27 (1.12, 1.44)***	1.49 (1.13, 1.96)**	50	6.08	1.65 (1.44, 1.89)***	1.60 (1.19, 2.16)**
Yes	198	9.52	39	11.4	1.19 (0.96, 1.49)	1.57 (1.11, 2.21)*	86	15.4	1.61 (1.37, 1.90)***	1.70 (1.32, 2.19)***

Rate^#^, incidence rate, per 1000 person-years; IRR*, incidence rate ratio; Adjusted HR^†^, multiple analysis including age, sex, and co-morbidities of CAD, alcohol-related illness, stroke, COPD, asthma, rheumatoid arthritis, medication of steroid used; Comorbidity^‡^: Only to have one of comorbidities (including CAD, alcohol-related illness, stroke, COPD, asthma, rheumatoid arthritis) classified as the comorbidity group

*p<0.05

**p<0.01

***p<0.001

After adjustment for age, sex, and comorbidities, the risk of developing osteoporosis was higher in the early *H*. *pylori* eradication cohort (HR = 1.52, 95% CI = 1.23–1.89) and late *H*. *pylori* eradication cohort (HR = 1.69, 95% CI = 1.39–2.05) than in the control cohort. The overall incidence and risk of osteoporosis were compared in the late *H*. *pylori* eradication cohort and control cohort according to several variables including age, sex, and comorbidities. The risk of osteoporosis in the late *H*. *pylori* eradication cohort was also higher than that of the control cohort. The risk of osteoporosis in patients receiving early *H*. *pylori* eradication therapy was also higher than that of the control cohort in all cases, except for those aged ≤49 years.

The risk of osteoporosis was compared in the early *H*. *pylori* eradication cohort and the late *H*. *pylori* eradication cohort according to several variables including age, sex, and comorbidities. The risk of osteoporosis in patients receiving early *H*. *pylori* eradication therapy was lower than that of the late eradication cohort; however, this difference was not statistically significant (Table 3).

**Table 3:** Hazard ratios of Osteoporosis between all gastric disease patients with early HP Eradication and with late HP Eradication stratified by demographic characteristics and comorbidity.

	Early HP Eradication		Late HP Eradication	
	IRR*	Adjusted HR^†^	IRR*	Adjusted HR^†^
	(95% CI)	(95% CI)	(95% CI)	(95% CI)
**All**	1 (Reference)	1 (Reference)	1.61 (1.38, 1.89)***	1.11 (0.85, 1.46)
**Gender**				
Female	1 (Reference)	1 (Reference)	1.44 (1.13, 1.84)**	1.11 (0.79, 1.57)
Men	1 (Reference)	1 (Reference)	1.81 (1.48, 2.22)***	1.02 (0.66, 1.58)
**Age**				
≤49	1 (Reference)	1 (Reference)	1.40 (1.09, 1.79)**	1.15 (0.59, 2.22)
≥50	1 (Reference)	1 (Reference)	1.28 (1.04, 1.57)*	1.10 (0.82, 1.48)
**Comorbidity**^**‡**^				
No	1 (Reference)	1 (Reference)	1.30 (1.07, 1.59)**	1.05 (0.72, 1.53)
Yes	1 (Reference)	1 (Reference)	1.35 (1.04, 1.77)*	1.09 (0.75, 1.60)

IRR*, incidence rate ratio; Adjusted HR^†^, multiple analysis including age, sex, and co-morbidities of CAD, alcohol-related illness, stroke, COPD, asthma, rheumatoid arthritis; Comorbidity^‡^: Only to have one of comorbidities (including CAD, alcohol-related illness, stroke, COPD, asthma, rheumatoid arthritis) classified as the comorbidity group

*p<0.05

**p<0.01

***p<0.001

For patients receiving early *H*. *pylori* eradication therapy, a 1.69-fold risk of developing osteoporosis was found within 5 years of follow-up (95% CI = 1.32–2.16) (Table 4). There was no statically significant difference between the control group and early *H*. *pylori* eradication group when the follow-up period was over 5 years. However, for patients receiving late *H*. *pylori* eradication therapy, higher risks were observed for developing osteoporosis in both follow-up periods (<5 y and >5 y; HR = 1.72, 95% CI = 1.38–2.14 and HR = 1.62, 95% CI = 1.06–2.47, respectively).

**Table 4:** Trends of Osteoporosis risks by stratified follow-up years.

	Control (N = 21788)		Early HP Eradication (N = 2721)		IRR *	Adjusted HR^†^	Late HP Eradication (N = 2726)		IRR*	Adjusted HR^†^
Follow time, years	Case	Rate^#^	Case	Rate^#^	(95% CI)	(95% CI)	Case	Rate^#^	(95% CI)	(95% CI)
≤5 years	405	4.62	75	6.67	1.44 (1.29, 1.62)***	1.69 (1.32, 2.16)***	109	10.3	2.23 (2.02, 2.46)***	1.72 (1.38, 2.14)***
>5 years	159	4.87	23	4.76	0.98 (0.82, 1.16)	1.14 (0.73, 1.77)	27	8.35	1.71 (1.46, 2.01)***	1.62 (1.06, 2.47)*

Rate^#^, incidence rate, per 1000 person-years; IRR *, incidence rate ratio; Adjusted HR^†^: multiple analysis including age, sex, and co-morbidities of CAD, alcohol-related illness, stroke, COPD, asthma, rheumatoid arthritis

*p<0.05

***p<0.001

## Discussion

The main finding of this population-based retrospective cohort study in Taiwan is that people who received *H*. *pylori* eradication therapy, those though to have chronic *H*. *pylori* infection, have a higher incidence of osteoporosis. The late eradication group, which had a longer period of chronic *H*. *pylori* infection, had a higher incidence of osteoporosis than the early eradication group. When the follow-up period was over 5 years, the incidence of osteoporosis among the early *H*. *pylori* eradication cohort returned to the levels observed in the control cohort, while the incidence in the late eradication group remained higher. We believe the higher incidence of osteoporosis within the first 5 years could be the result of chronic *H*. *pylori* infection before *H*. *pylori* eradication therapy, and people could benefit from early eradication therapy when the follow-up period is greater than 5 years.

The NHIRD covers almost 99% of the population of Taiwan and is a representative data source that includes age, sex, and comorbidity information. It enabled this study—the first of its type nationwide—to be conducted with population-based data and a near-decade follow-up period to date. Although our results do not directly indicate the causal relationship and etiological treatise for this association, based on literature review, we believe that there are several possible explanations.

Chronic *H*. *pylori* infection may be the reason for the increased incidence of osteoporosis observed after eradication therapy. First, chronic *H*. *pylori* infection may alter calcium absorption. *H*. *pylori* can either increase gastric acid production, which predisposes one to duodenal ulceration, or decrease gastric acid production, thus leading to pangastritis or gastric ulceration [15, 30]. The hypochlorhydric stomach impairs calcium homeostasis and alters bone mass [31, 32]. The chronic gastritis from *H*. *pylori* results in atrophic gastritis, which increases the likelihood of osteoporosis [10, 33]. Second, a recent population-based cohort study in Taiwan suggested that chronic *H*. *pylori* infection increased the subsequent risk of end-stage renal disease (ESRD) [17]. Vitamin D, which is crucial in the gastrointestinal absorption of calcium, is commonly insufficient in patients with chronic kidney disease and ESRD [34, 35]. Furthermore, Ayesh et al. found that people with *H*. *pylori* infection had lower levels of vitamin B12 [36]. In addition, low plasma levels of vitamin B12 may be associated with low bone mineral density [37].

*H*. *pylori* infection may cause chronic systemic inflammation that negatively affects bone mineral density. *H*. *pylori* is the most common chronic bacterial infection of the human upper gastrointestinal tract [25, 38], and causes an increase in the serum level of inflammatory cytokines [39, 40]. An elevated serum level of inflammatory cytokines is considered to be associated with an increased risk of osteoporosis [41]. This is consistent with the increased incidence of osteoporosis among the *H*. *pylori* eradication groups in our study. Early eradication of *H*. *pylori* may prevent chronic inflammation and the subsequent prolonged elevation of serum levels of inflammatory cytokines that lead to osteoporosis, as described by Moss et al. in 1994 [42].

**Fig 1:**
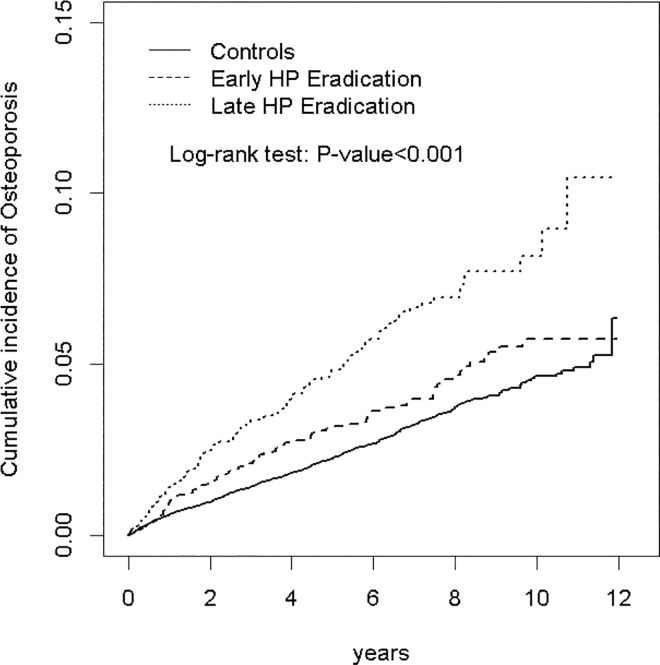
Cumulative incidence of Osteoporosis for patients with gastric disease receiving early HP Eradication and Late HP Eradication compared to those without gastric disease.

We believed that the increased incidence of osteoporosis among eradication groups could be the result of chronic *H*. *pylori* infection before eradication therapy, rather than the effect of eradication therapy. If it were the effect of eradication therapy, the early eradication group might exhibit a higher incidence of osteoporosis, especially in the early years after therapy. In contrary, although non-significant, we observed a lower incidence of osteoporosis among the early *H*. *pylori* eradication group compared with the late *H*. *pylori* eradication group (Table 3). The early eradication group had a lower incidence of osteoporosis than the late eradication group among first five years after eradication therapy (Table 4). These were against the hypothesis that the eradication therapy would increase incidence of osteoporosis. As shown in Table 4 and Fig 1, the incidence of osteoporosis among the early eradication group returned to the same level as that of the control group over the course of long-term follow-up, whereas the late eradication group’s incidence rate remained high. These could be the evidence that eradication therapy may eliminate chronic inflammation from *H*. *pylori* infection before treatment, which decreased the *H*. *pylori* related influence on bone density.

*H*. *pylori* infection is associated with several gastrointestinal diseases, and the medication used in gastrointestinal diseases may be another cause of osteoporosis. Malabsorption of dietary calcium is a cause of osteoporosis, and gastric acid is necessary in calcium absorption [43]. The use of proton pump inhibitors and H2 receptor blockers in *H*. *pylori* eradication therapy can block the secretion of gastric acid that decrease calcium salt absorption, leading to osteoporosis. A meta-analysis by Ngamruengphong et al. demonstrated that using a proton pump inhibitor increased the risk of hip and vertebral fractures [44]. In the current study, we found a higher incidence of osteoporosis in the *H*. *pylori* eradication groups. This mechanism treatise could correlate with gastrointestinal calcium malabsorption from *H*. *pylori* eradication therapy. However, it still requires further approaches to clarify the causal relationship and detailed mechanism.

This study has several limitations. First, the NHIRD does not disclose patients’ socioeconomic status, family history, personal health behaviors (e.g., smoking habits or alcohol consumption), laboratory data, or biomarkers. Osteoporosis could be affected by these confounding factors, and may have influenced our results. Second, the NHIRD database recorded only patients who received therapy for *H*. *pylori* infection and osteoporosis. With symptoms that are difficult to detect, the prevalence of both *H*. *pylori* infection and osteoporosis could be underestimated. Third, the severity and duration of *H*. *pylori* infection could not be assessed in this study. Fourth, we cannot identify the method used to diagnose osteoporosis and the intensity of osteoporosis in the NHIRD database. Finally, the exact mechanism between *H*. *pylori* eradication therapy and osteoporosis could not be identified via a retrospective cohort study using the NHIRD.

## Conclusion

In this study, we found a higher incidence of osteoporosis among people with *H*. *pylori* infection. Early eradication of *H*. *pylori* had a relatively lower incidence of osteoporosis when compared with the late eradication group. When the follow-up period was over 5 years, there was no difference between the control group and early *H*. *pylori* eradication group. This provides evidence that encourages physicians to manage *H*. *pylori* infections early in patients with a high risk of osteoporosis. Additional studies are necessary to clarify the relationship between *H*. *pylori* infection and eradication, and osteoporosis, after adjustment for confounding factors, and to identify the mechanism of the relationship between *H*. *pylori* and osteoporosis.

## Abbreviations

aHR: adjusted hazard ratio
CI: confidence interval
ICD-9-CM: International Classification of Diseases, Ninth Revision, Clinical Modification
NHIRD: National Health Insurance Research Database
LHID 2000: Longitudinal Health Insurance Database 2000
